# Association of Obesity, Sarcopenia, and Sarcopenic Obesity With Hypertension in Adults: A Cross-Sectional Study From Ravansar, Iran During 2014–2017

**DOI:** 10.3389/fpubh.2021.705055

**Published:** 2022-02-02

**Authors:** Yahya Pasdar, Mitra Darbandi, Shahab Rezaeian, Farid Najafi, Behrooz Hamzeh, Amir Bagheri

**Affiliations:** ^1^Research Center for Environmental Determinants of Health (RCEDH), Health Institute, Kermanshah University of Medical Sciences, Kermanshah, Iran; ^2^Social Development and Health Promotion Research Center, Kermanshah University of Medical Sciences, Kermanshah, Iran; ^3^Cardiovascular Research Center, Kermanshah University of Medical Sciences, Kermanshah, Iran; ^4^Department of Community Nutrition, School of Nutritional Sciences and Dietetics, Tehran University of Medical Sciences, Tehran, Iran; ^5^School of Nutrition Sciences and Food Technology, Kermanshah University of Medical Sciences, Kermanshah, Iran

**Keywords:** sarcopenia, obesity, sarcopenic obesity, body composition, hypertension, blood pressure

## Abstract

**Background and Aims:**

Hypertension may lead to disability and death by increasing the risk of cardiovascular disease, kidney failure, and dementia. This study aimed to determine the association between obesity, sarcopenia and sarcopenic obesity, and hypertension in adults resident in Ravansar, a city in the west of Iran.

**Methods:**

This cross-sectional study was conducted on 4,021 subjects from the baseline data of the Ravansar Non-Communicable Disease (RaNCD) cohort study, in the west region of Iran, from October 2014 up to February 2017. Body composition was categorized into obese, sarcopenia, sarcopenic obese, and normal based on measurements of muscle strength, skeletal muscle mass, and waist circumference. Univariate and multiple logistic regression models were used to examine the relationships, using the STATA 15 software.

**Results:**

The mean age of the participant was 47.9 years (SD: 8.4), the body mass index (BMI) was 26.84 kg/m^2^ (SD: 4.44), and the prevalence of hypertension was 15.12%. The prevalence of obesity, sarcopenia, and sarcopenic obesity were 24.37, 22.01, and 6.91%, respectively. Body composition groups had significant differences in age, total calorie intake, BMI, skeletal muscle mass, and muscle strength (*P*-value ≤ 0.001). In crude model, the obese (OR = 2.64; 95% CI: 2.11–3.30), sarcopenic (OR = 2.45; 95% CI: 1.94–3.08), and sarcopenic obese (OR = 3.83; 95% CI: 2.81–5.22) groups had a higher odds of hypertension. However, in adjusted models, only the obese group had a higher likelihood of hypertension (OR = 2.18; 95% CI: 1.70–2.80).

**Conclusion:**

This study showed that obesity was associated with hypertension, whereas sarcopenia and sarcopenic obesity had no significant relationship with hypertension.

## Introduction

Hypertension is the main cause of death or disability in the world (1). In comparison to developed countries, the risk of deaths from hypertension is more than doubled in low and middle-income countries for all ages (2, 3). A systematic review showed that the prevalence of hypertension in the Iranian population was 22.1% (4). Alcohol consumption, physical inactivity or unhealthy diet, body size, or body composition might be risk factors for hypertension (5, 6).

Muscle performance and skeletal muscle mass continuously decrease during aging. Sarcopenia refers to a condition that the decline in muscle mass and muscle function is more than regular age-dependent progress (7, 8). Sarcopenia is also known as an important component of fragility that is associated with a physical disability, the tendency to fall, mortality, inflammation, and insulin resistance (9–13). Despite cross-sectional studies showing that sarcopenia was significantly associated with odds of hypertension (14, 15), a prospective cohort study did not confirm such a relationship for cardiovascular diseases (16).

Aging, which is accompanied by a decrease in physical activity, is not only related to reductions in muscle mass but also could increase the fat mass (12). As age increases, fat distribution changes in the body, which is associated with increased visceral fat, as well as fat depositions that happen in the liver, heart, skeletal muscle, and pancreas (17). Obesity, especially the fat stored in visceral tissue, produces extra pro-inflammatory adipokines, which leads to a low-grade inflammatory state (18). This low-grade inflammatory disease can lead to a loss of skeletal muscle mass, cognitive decline, a decrease in immune function, increased insulin resistance, and atherosclerosis (19–21). Moreover, studies revealed that the prevalence of hypertension was significantly higher in people with obesity than non-obese subjects (22).

Sarcopenic obesity represents a combination of sarcopenia and obesity, which means unusual muscle loss, coinciding with fat accumulation (23). Studies suggest that when obesity and muscle loss co-exist, they can synergistically increase the risk of several diseases (23, 24). According to cohort studies conducted in Korea (14, 25) and the United States (26), sarcopenic obesity was related to increasing the risk of hypertension. However, dos Santos et al. found that sarcopenia and sarcopenic obesity were not associated with cardiometabolic impairments (27). Due to the heterogeneity between studies and limited evidence on the relationship between sarcopenic obesity and hypertension in different societies, especially in Iran, the main purpose of this study was to examine which body composition indices including obesity, sarcopenia, and sarcopenic obesity were associated with the odds of hypertension according to an assessment of both muscle strength and muscle mass in adults resident in Ravansar, a city in the west of Iran.

## Materials and Methods

### Study Population

The study was carried out as a cross-sectional analysis of the baseline information from the Ravansar Non-Communicable Diseases (RaNCD). The comprehensive information on the setting, location, data collection, and sampling method procedure have been published previously (28, 29). In total, the RaNCD cohort was a population-based study with aim of investigating the non-communicable diseases in Kurdish participants in Ravansar city, Kermanshah Province, west of Iran. Ravansar is a district with urban and rural areas, located in the west of Iran in the province of Kermanshah with a population of about 50,000 people, all of the Iranian Kurdish ethnicity. This cohort was one of the ten centers of the Prospective Epidemiological Research Studies in Iran (PERSIAN) mega cohort study that is approved by the ethics committees at the Ministry of Health and Medical Education, Tehran University of Medical Sciences, Iran. Baseline data were collected from October 2014 up to February 2017, and 10,000 adults between the ages of 35 and 65 (both men and women), who were registered as permanent residents of Ravansar were included in this cohort (28). In the present study, all participants in the baseline phase of the RaNCD cohort study were included in the study.

**Figure 1:**
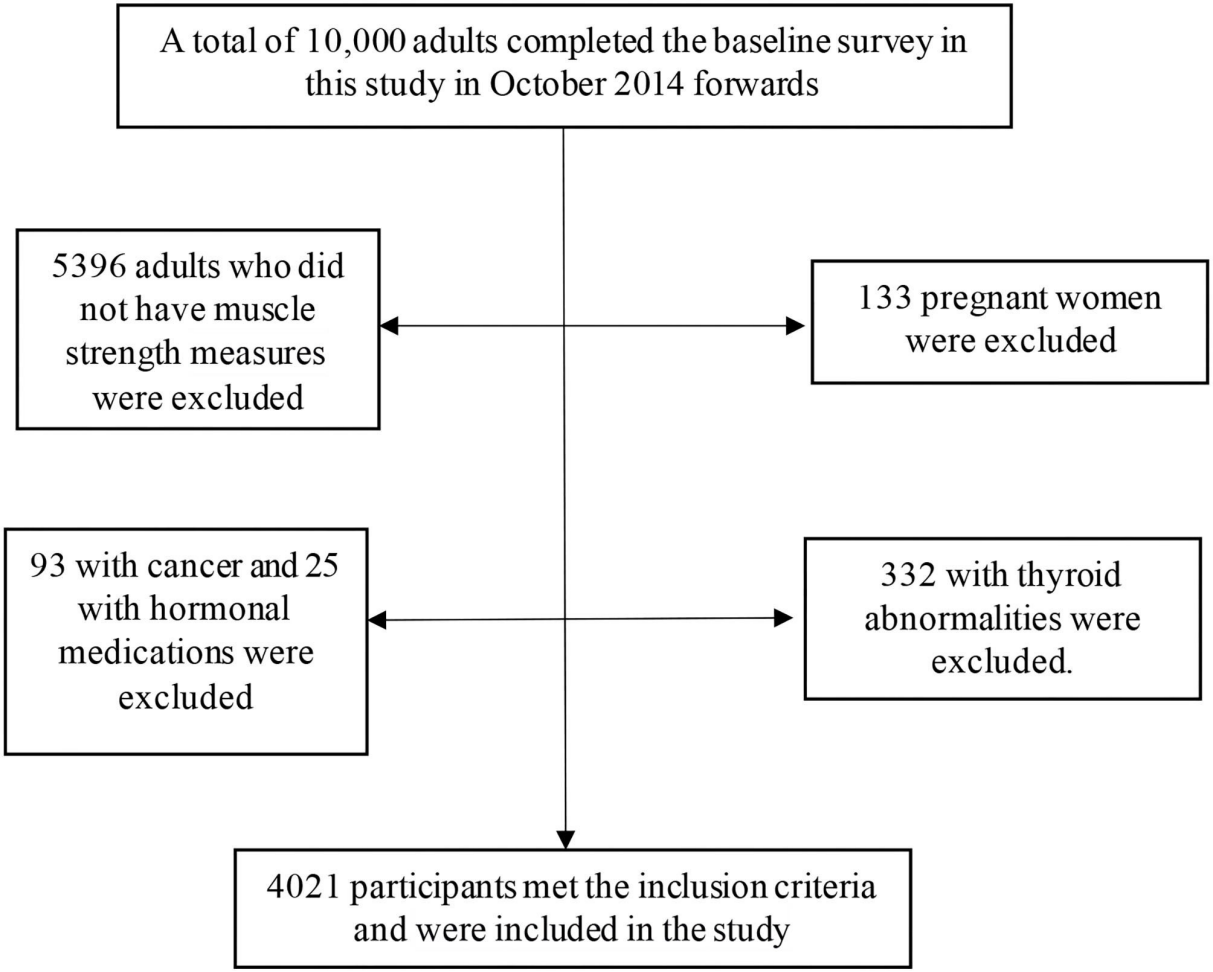
Flowchart of the present study.

The inclusion criteria for the participants in the RaNCD cohort study were (A) participants were permanent residents of Ravansar; (B) within the age range of 35–65 y; (C) inclination to participate in the study along with the possibility of staying in Ravansar for the upcoming future; (D) provided written informed consent. In the RaNCD study, the subjects that were reluctant to participate in the study lived in Ravansar for less than one year and were unable to come to the cohort center or to communicate with interviewers (due to mental or physical disability, blindness, deafness, unable to speak, and affected by any acute psychological disorder) were not included. In the current cross-sectional study, participants were excluded according to the following exclusion criteria: 5,396 subjects without muscle strength measures, 133 pregnant women, 93 with cancer, 25 with hormonal medications, and 332 with thyroid abnormalities. Finally, 4,021 participants met the inclusion criteria and were included in the study (Figure 1).

### Data Collection

All the individuals who participated in the study were examined through telephone and face-to-face interviews and clinical examinations. The data collection and management were carried out by using database applications. For example, all questionnaires are filled out through an online survey and regularly supervised by internal and external evaluation teams. Questionnaire information was collected by well-trained staff through face-to-face interviews. Information about age, sex, marital status, education, economic status, physical activity, history of smoking, alcohol consumption, and history of chronic diseases was recorded online in an electronic questionnaire form. Dietary data were collected through 125-item food frequency questioners (28). Weight, height, waist circumference (WC), and hip circumference were measured by trained researchers. The PERSIAN Cohort standard physical activity questionnaire was used to assess participants' physical activity. The questionnaire consisted of 22 items about the different activities of the participants during the day. Then, physical activity was categorized into three groups (light, moderate, and high) based on activity intensity. The details of the standard protocols of measurements have been published in the study of the RaNCD cohort profile (28).

### Body Composition

All contributors were asked to remove their heavy clothes, shoes, and accessories. Then, body composition including body weight, body mass index (BMI), and skeletal muscle mass (SMM) were measured using an automated Bio-Impedance Analyzer BIA (Inbody 770, Inbody Co, Seoul, Korea) with a precision of 0.5 kg (28, 30). Height was measured by an automatic BSM 370 (Biospace Co., Seoul, Korea) with a precision of 0.1 cm. All tools and devices in the RaNCD cohort center were calibrated before the study began (28).

To measure abdominal obesity, WC was selected as an indicator of obesity, because abdominal obesity is a strong predictor of risk factors for cardiovascular disease and hypertension (31). WC was measured by non-stretched and flexible tape in the narrowest area, at the distance between the last ribs and the top of the iliac crest when the subjects were in the expiratory state. Studies indicated that WC has a high correlation with total, abdominal, and visceral fat (32).

### Handgrip Strength

To measure muscle strength, handgrip strength was measured, using a digital dynamometer (Seahan, model SH5003, Seahan Co, South Korea). Muscle strength was measured based on the Southampton protocol. According to this protocol, the participants performed the test using the dominant hand in a sitting position on a chair, with their elbow extended to 90° along the vertical axis and their wrists in slight extension. This test was repeated 3 times with 15 s between trials, and the mean value was recorded in kilogram. The calibration of this dynamometer was conducted based on the manufacturers' manual. The calibration for the device was performed at the factory by loading it at the center with weights and making appropriate adjustments in the gauge. This process for the calibration should be done once a year. If there was an error in the calibration, the device should be returned to SAEHAN Corporation for recalibration. The validity and reliability of the dynamometer were confirmed previously (33). Grip strength was chosen as an indicator of overall muscle strength, because of its excellent reproducibility. There is also evidence that grip strength is highly correlated with the strength of other muscle groups (34).

### Definitions of Sarcopenia

Sarcopenia was categorized based on skeletal muscle mass and skeletal muscle strength. Initially, individuals were divided into sex-specific tertile (low, moderate, and high), based on WC, muscle strength, and muscle mass. Individuals with low or moderate WC tertiles and moderate or high muscle mass and muscle strength tertile were categorized as having a “normal” body composition. Individuals who were classified as “obese” were in the high WC tertiles and had either moderate or high muscle mass and muscle strength tertiles. Individuals were classified as “sarcopenic” if they had either the low or moderate WC tertile and the low muscle mass and low muscle strength tertile. Lastly, individuals who were categorized as “sarcopenic obese,” were in the high tertile of WC and the low tertile of muscle mass and muscle strength (34).

### Blood Pressure Measurement

To measure the blood pressure, people were asked to sit on a chair, and blood pressure was measured by a manometer (Rudolf Riester GmbH, Bruckstr, Jungingen, Germany) cuff and stethoscope (Rudolf Riester GmbH, Bruckstr, Jungingen, Germany), in the seated position and from both right and left arm, and it was repeated 10 min later. The blood pressure of all the participants was measured in the morning to minimize the effects of diurnal variation. The mean of the readings was recorded as the final blood pressure. Blood pressure was coded as normal (<120/80 mmHg), pre-hypertensive (120/80–139/89 mmHg), or hypertensive (140/90 mmHg and/or medication use) (25).

### Statistical Analysis

Continuous variables are presented as mean ± SD and categorical variables are presented as *n* (%). The normality test was checked using the Kolmogorov–Smirnov test for the continuous variables. ANOVA test was used to compare the mean values among the four groups and the Scheffe *post-hoc* test was also applied to determine the significant difference between the groups. To examine the association between hypertension and sarcopenia, obesity, or sarcopenic obesity, univariate and multiple logistic regression models were used to estimate crude and adjusted odds ratios (ORs), and 95% CIs, respectively. In model 1, the relationship was controlled for age (years) and sex (male/female). In the second model, additional adjusting was applied for alcohol (yes/no), total calorie (kcal/d), carbohydrate (g/d), total fat (g/d), place (rural/urban), quantiles of education, quantiles of wealth, and physical activity (MET-h/wk). Missing was controlled by the imputation method. All of the statistical analyses were analyzed using STATA software version 15(StataCorp, Lakeway Drive College Station, Texas, USA). The significance level was set at a *P*-value < 0.05.

## Results

Out of 10,000 participants in the Ravansar cohort, 4,021 participants met the inclusion criteria. Table 1 shows the characteristics of participants. The mean age of the participants was 47.9 years (SD: 8.4), the total calorie intake was 3,177 kcal per day (SD: 1,100), and the BMI was 26.84 kg/m^2^ (SD: 4.44). In addition, 55.7% of participants were men, 75.2% were living in a village, 25.7% were smoking, 7.5% used alcohol, and 15.1% of them had hypertension. The other characteristics, such as quantiles of wealth, education levels, and physical activity were shown in Table 1.

**Table 1:** Characteristics of participants in this study.

**Variables**	Mean ±SD; *n* (%)^*^
Age (years)	47.9 ± 8.4
Total calorie (kcal/d)	3,177 (,1100)
Body mass index (kg/m^2^)	26.84 ± 4.44
**Gender**
Male	2,240 (55.71)
Female	1,781 (44.29)
**Quantiles of wealth**
1 (Poorest)	910 (22.63)
2	879 (21.86)
3	843 (20.96)
4	756 (18.80)
5 (Richest)	633 (15.74)
**Education years**
Illiterate	1,096 (27.26)
1–5 years	1,438 (35.76)
6–9 years	709 (17.63)
10–12 years	520 (12.93)
>13	258 (6.42)
**Physical activity**
High	1,065 (26.49)
Moderate	1,892 (47.05)
Light	1,064 (26.46)
**Place**
City	995 (24.75)
Village	3,026 (75.25)
**Smoking**
Yes	1,035 (25.74)
No	2,986 (74.26)
**Alcohol use**
Yes	303 (7.54)
No	3,718 (92.46)
**Hypertension**
Yes	608 (15.12)
No	3,413 (84.88)

* *Data are presented as mean ± SD for continuous variables and frequency (%) for categorical variables*.

The baseline characteristics of individuals according to sarcopenia, obesity, and sarcopenic obesity classification are indicated in Table 2. Out of the 4,021 included subjects, 1,878 (46.7%) were classified as normal, 885 (22.01%) had sarcopenia, and 980 (24.3%) of them had obesity. Furthermore, 278 (6.9%) of them had sarcopenic obesity. Based on one-way ANOVA, there were significant differences between body composition groups and age, total calorie intake, carbohydrate intake, protein intake, BMI, skeletal muscle mass, and muscle strength (*P*-value ≤ 0.001). The Scheffe *post-hoc* test was conducted to establish the differences between body composition groups (Table 2). Accordingly, the normal group was significantly younger, had lower BMI, lower WC, and had higher skeletal muscle mass and muscle strength than the obese, sarcopenia, and sarcopenic obesity groups (*P*-value ≤ 0.001). Also, the obese group was younger (*P*-value ≤ 0.001), had higher calorie intake (*P*-value ≤ 0.001), had higher WC, and had higher skeletal muscle mass and muscle strength (*P*-value ≤ 0.001) than the sarcopenia and sarcopenic obesity groups. Finally, the sarcopenic group was younger, had lower BMI, and had lower WC and skeletal muscle mass than the sarcopenic obesity group (*P*-value ≤ 0.001).

**Table 2:** Baseline characteristics of groups according to sarcopenia and obesity classification.

**Variables**	**Normal**	**Sarcopenia**	**Obese**	**Sarcopenic-obese**
Number (%)	1,878 (46.7)	885 (22.01)	980 (24.37)	278 (6.91)
Age (year)^*^	45.871 (7.7)	51.254 (9.01)^a^^b^^c^	47.637 (7.6)^a^	52.672 (8.2)^a^^b^
Total calorie (kcal/d)^*^	3478.2 (1072.8)	2532.4 (885.6)^a^^b^	3369.3 (1068)	2520.2 (854.3)^a^^b^
Carbohydrate (g/d)^*^	402.2 (145.7)	401.4 (4)	411.8 (148.4)^a^	406.5 (151.3)^a^^b^
Total fat (g/d)^*^	78.5 (33.8)	79.2 (35.2)	79.3 (32.9)	79.2 (34.1)
Total protein (g/d)^*^	91.1 (36.6)	90.45 (38.8)^b^	93.3 (36.8)^a^	91.8 (37.8)
BMI (kg/m^2^)^*^	25.007 (3.1)	24.42 (3.3)^a^^b^^c^	31.376 (3.6)^a^	30.916 (2.89)^a^
Waist circumference (cm)^*^	93.3 (6.07)	90.9 (7.6)^a^^b^^c^	109 (5.2)^a^	107.5 (4.3)^a^^b^
Skeletal muscle mass (kg)^*^	29.3 (3.8)	19.3 (2.01)^a^^b^^c^	30.5 (5.2)^a^	20.6 (1.3)^a^^b^
Muscle strength (kg)^*^	39.8 (8.9)	20.01 (3.3)^a^^b^	37.4 (9.6)^a^	19.8 (3.06)^a^^b^

* *Data are presented as mean ± SD*.

a *P-value < 0.001 for sarcopenia, obese, sarcopenic-obese vs. normal group*.

b *P-value < 0.01 for sarcopenia and sarcopenic-obese vs. obese group*.

c *P-value < 0.001 for sarcopenia vs. sarcopenic-obese group*.

*BMI, Body Mass Index; kg, kilogram; kcal, kilocalorie; g, gram; d, day*.

The OR and CI for hypertension, according to sarcopenia, obesity, and sarcopenic obesity classifications are shown in Table 3. The crude model shows that sarcopenia (OR: 2.45; 95% CI: 1.94–3.08), obesity (OR: 2.64; 95% CI: 2.11–3.30), and sarcopenic obesity (OR: 3.83; 95% CI: 2.81–5.22) increased the odds of hypertension. Model 1, adjusted for age and sex shows that only the obese group was significantly related to hypertension (OR: 2.29; 95% CI: 1.79–2.93). Whereas, sarcopenia (OR = 1.01; 95% CI: 0.71–1.45) and sarcopenic obese groups (OR = 1.5; 95% CI: 0.98–2.30) were not associated with hypertension. Model 2 adjusted for age, sex, alcohol, total calorie, carbohydrate, total fat, place, education, quantiles of wealth, and physical activity, shows that only the obese group had a higher odds of hypertension (OR = 2.18; 95% CI: 1.70–2.80). However, no significant relationship was observed between sarcopenia (OR = 0.95; 95% CI:0.66–1.36) and sarcopenic obese groups (OR = 1.39; 95% CI:0.90–2.14) and OR of hypertension.

**Table 3:** Odds ratios and confidence intervals for hypertension according to sarcopenia and obesity classification.

	**Normal**	**Sarcopenia**	**Obese**	**Sarcopenic-obese**
Crude	1 (Reference)	2.45 (1.94–3.08)^a^	2.64 (2.11–3.30)^a^	3.83 (2.81–5.22)^a^
Model 1	1 (Reference)	1.01 (0.71–1.45)	2.29 (1.79–2.93)^a^	1.5 (0.98–2.30)
Model 2	1 (Reference)	0.94 (0.65–1.35)	2.15 (1.67–2.77)^a^	1.35 (0.87–2.09)

*Model 1, Adjusted for age and sex*.

*Model 2, Adjusted for age, sex, alcohol, total calorie (kcal), carbohydrate (g), total fat (g), place, education, quantiles of wealth, physical activity*.

a *P-value < 0.001*.

## Discussion

The main target of this study was to clarify the relevance of obesity, sarcopenia, and sarcopenic obesity for the odds of hypertension in adult residents in the west of Iran. This study showed that only the obese group was associated with the odds of hypertension after adjusting for main confounders, whereas the sarcopenia and sarcopenic obese groups had no significant relationship with hypertension.

Based on the result, obesity was positively associated with hypertension. In line with our findings, a study on obesity and cardiovascular risk factors including hypertension in Americans showed that people with obesity had a significantly higher prevalence of hypertension than groups without obesity (35). Moreover, a cross-sectional study in Northwest Ethiopia has demonstrated that the relationship between hypertension and obesity could be different in distinct countries. Accordingly, it showed that in Northwest Ethiopia, the prevalence of hypertension is lower than in Uganda, Mozambique, Eastern Nigeria, and Northern India, and this difference may be due to the higher prevalence of obesity (22). However, we failed to find a significant relationship between sarcopenia and sarcopenic obesity and odds of hypertension. In line with our survey, in a prospective study conducted on 4,252 adult men in England (16), investigators indicated that there was no significant association between sarcopenia and sarcopenic obesity with risk of CVD and CHD events. Similarly, another cohort study conducted on 3,366 older adults in the United States (34), showed that sarcopenic obesity identified based on muscle mass was not significantly related to cardiovascular disease (CVD). Nevertheless, some studies showed that sarcopenia and sarcopenic obesity had a significant relationship with hypertension (14, 15, 25). It seems that obesity is significantly related to hypertension and the conflicting findings on the association between sarcopenic obese subjects and the risk of hypertension, may be due to differences in the study populations, and the lack of a single diagnostic method or different tools of body composition assessment that have been used to diagnose sarcopenic obesity. Therefore, more studies in this regard are suggested.

Obesity can induce hypertension through various mechanisms. First, increasing leptin leads to hypertension *via* increasing the sympathetic nervous system (SNS) (36, 37). Second, low serum levels of adiponectin cause endothelial dysfunction and hypertension through increasing insulin resistance (38, 39). Third, the high level of thromboxane A2 (TXA2), plasminogen activator inhibitor-1 (PAI-1), inflammation factors (IF), free-fatty acids (FFA), and angiotensinogen (AGT) may be related to hypertension (40). It should be noted that central obesity, as well as visceral adipose tissues (VATs), are directly related to hypertension (41). Because in comparison with total adiposity, VATs are associated with elevated inflammatory cytokines, insulin resistance, atherosclerosis, and cardiovascular problems (41).

The present study has several strengths. The large sample size is a strength of this study. It is also the first study that has been conducted on the Iranian Kurdish ethnic group. Therefore, it could be an appropriate reference for future studies that will be conducted on other ethnicities since it provides the possibility of comparisons between ethnicities. Despite these strengths, our study has some limitations. The cross-sectional design is one of the main limitations of this study that prevents us from identifying causal relationships. Another study limitation is that BIA is not a gold-standard tool for the measurement of body composition; however, this tool is reasonably accurate for use in large studies. Finally, the study was conducted on the Kurdish population and therefore, extrapolation of the present findings to other ethnic groups might not be done. Thus, a well-design prospective cohort study on a large population and different racial groups is recommended.

## Conclusion

In conclusion, this study showed that obesity was significantly linked with hypertension; however, we failed to find a significant association between sarcopenia and sarcopenic obesity and odds of hypertension.

## Data Availability Statement

The raw data supporting the conclusions of the article will be made available by the authors, on reasonable request to the corresponding author.

## Ethics Statement

The studies involving human participants were reviewed and approved by the Research and Technology Deputy and the Ethical Committee of Kermanshah University of Medical Sciences. The patients/participants provided their written informed consent to participate in this study.

## Author Contributions

YP, FN, AB, and BH contributed to the planning of the study. Statistical analyses were completed by SR and MD. AB controlled data quality and wrote the paper. All authors contributed to the interpretation of results, editing of the manuscript, read, and approved the final manuscript.

## Funding

This study was supported by Kermanshah University of Medical Sciences (grant number: 94091) for financial support.

## Conflict of Interest

The authors declare that the research was conducted in the absence of any commercial or financial relationships that could be construed as a potential conflict of interest. The reviewer MP declared a shared affiliation with one of the authors, AB, to the handling editor at time of review.

## Publisher's Note

All claims expressed in this article are solely those of the authors and do not necessarily represent those of their affiliated organizations, or those of the publisher, the editors and the reviewers. Any product that may be evaluated in this article, or claim that may be made by its manufacturer, is not guaranteed or endorsed by the publisher.
